# A deep learning-based silhouette gait detection framework for fall risk classification in Parkinson’s disease

**DOI:** 10.3389/fneur.2026.1944666

**Published:** 2026-09-29

**Authors:** Slo-Li Chu, Chia-Ju Kuo, Chun-Yen Chiu, Chiaching Wang, Shey-Lin Wu, Ping-Yi Lin

**Affiliations:** 1 Department of Information and Computer Engineering, Chung Yuan Christian University, Taoyuan, Taiwan; 2 Department of Neurology, Show Chwan and Chang Bing Show Chwan Memorial Hospitals, Changhua, Taiwan; 3 Department of Nursing, Hungkuang University, Taichung, Taiwan; 4 Department of Medical Research, China Medical University Hospital, Taichung, Taiwan

**Keywords:** deep learning, fall risk classification, gait assessment, Parkinson’s disease, silhouette gait detection

## Abstract

**Background:**

Parkinson’s disease (PD) is a rapidly growing neurodegenerative disorder associated with gait impairment, functional decline, and increased fall risk. Conventional clinical assessments rely heavily on brief observational scales and are limited by subjectivity and environmental bias. There is a pressing need for objective, quantitative, and scalable digital assessment tools to support precision management of PD.

**Objective:**

This study aimed to develop and validate a smartphone-video-based gait assessment system aligned with a clinically meaningful pathway encompassing PD screening, functional evaluation, and fall risk classification.

**Methods:**

In this cross-sectional observational study, patients with mild-to-moderate PD and age-matched healthy controls were recruited following Institutional Review Board approval. Gait videos were recorded using smartphone cameras and processed through a silhouette extraction pipeline. Spatiotemporal gait features were subsequently extracted using a MoviNet-based deep learning framework. A multi-task framework was developed to perform PD screening, activities of daily living functional grading based on the Schwab and England Activities of Daily Living (S&E ADL) scale, and fall risk classification. Model performance and generalizability were evaluated using subject-independent data partitioning and independent cross-site validation.

**Results:**

A total of 40 participants were recruited. For PD screening, the model achieved an accuracy of 95.7%, a sensitivity of 96.6%, a specificity of 92.2%, and an AUC of 0.994 on the independent test set. For S&E ADL functional grading, the model achieved an overall accuracy of 88.5% across three functional categories (80, 90, and 100%). For fall risk classification, the model achieved an accuracy of 91.1%, a sensitivity of 68.4%, a specificity of 98.3%, and an AUC of 0.909. Overall, the framework demonstrated robust performance across PD screening, functional stratification, and fall risk classification tasks.

**Conclusion:**

This study developed a smartphone-video-based gait assessment framework using computer vision and deep learning. The proposed framework demonstrated strong performance in PD identification, severity estimation, and fall risk classification. Future work will expand validation across diverse populations and disease stages, while advancing toward multimodal integration and long-term home monitoring.

## Introduction

Population aging has become a major global public health challenge, accompanied by a rapid increase in age-related neurological disorders. Recent epidemiological studies indicate that neurological conditions have become a leading contributor to the Global Burden of Disease. It is estimated that in 2021, approximately 3.4 billion people, accounting for 43.1% of the global population, were affected by neurological conditions (1). Among these, Parkinson’s disease (PD) is recognized as one of the fastest-growing neurodegenerative disorders. Its prevalence continues to rise alongside population aging, imposing a heavy burden on healthcare systems, caregivers, and society as a whole (2). Parkinson’s disease is a progressive neurodegenerative disorder primarily characterized by tremor, rigidity, bradykinesia, and postural instability (3, 4). Early and accurate diagnosis is crucial for effective disease management, as timely intervention can slow functional decline and improve quality of life (5). However, early motor symptoms of PD, such as subtle gait instability or slight postural changes, are often difficult to detect during routine clinical examinations (6). Current clinical assessment tools, including the Unified Parkinson’s Disease Rating Scale (UPDRS), rely heavily on clinician observation and subjective judgment. Although the UPDRS remains the current gold standard, its observational nature is inherently susceptible to inter-rater variability and environmental bias (3). Consequently, there is an urgent need for objective, quantitative, and scalable digital assessment tools to support the precision management of Parkinson’s disease.

### Gait analysis as a digital biomarker

Gait is one of the most fundamental human motor activities and serves as a critical indicator of neurological function. A growing body of evidence suggests that gait abnormalities are often early manifestations of neurodegenerative diseases and are closely linked to cognitive decline, functional impairment, and an increased risk of falls (7). Traditional gait recognition has predominantly utilized wearable tracking sensors to monitor structural mobility metrics such as velocity, step progression, and postural orientation (8). Consequently, contemporary frameworks favor markerless, vision-based methodologies that capture dynamic locomotion data remotely via digital camera sensors (9, 10). Clinical Gait Analysis (CGA) enables the objective assessment of motor function by quantifying “spatiotemporal parameters,” providing more reliable information than traditional visual observation (11). With significant breakthroughs in deep learning and computer vision, utilizing consumer-grade imaging devices (such as cameras and smartphones) to capture gait footage for quantifying PD symptoms has emerged as a highly promising research direction (12–14). Recent studies have further demonstrated that human pose estimation can extract clinically meaningful gait information from conventional videos. Stenum et al. showed that video-derived estimates of step time, step length, and walking speed corresponded well with three-dimensional motion capture measurements, supporting the feasibility of RGB video for clinical gait analysis (15). Moreover, spatiotemporal movement patterns extracted from video data have been shown to reflect the severity of PD motor symptoms, and previous studies have demonstrated the feasibility of estimating MDS-UPDRS gait scores from gait videos (16, 17). For example, Zeng et al. combined skeletal and silhouette features to estimate MDS-UPDRS gait scores and distinguish patients with PD from healthy controls (18). Tian et al. further utilized spatiotemporal relationships among skeletal joints across consecutive frames to estimate MDS-UPDRS gait severity (19, 20). More recently, Han et al. demonstrated that lateral-view gait videos captured using smartphones could be used to assess PD gait impairment and detect subtle gait changes associated with medication intervention (12). Collectively, these findings suggest that spatiotemporal movement features derived from conventional gait videos have potential value as digital biomarkers for objective PD assessment.

Existing video-based gait analysis approaches commonly use human pose estimation to represent gait as skeletal keypoints or skeletal images. Skeletal representations explicitly capture joint locations and body structure, facilitating the analysis of joint-level movement patterns. In contrast, silhouette-based representations preserve more complete information on body contour, posture, limb configuration, and their dynamic changes across consecutive frames while reducing background, clothing color, and other appearance-related information. However, silhouettes do not directly provide joint angles, joint trajectories, or detailed kinematic parameters. Therefore, silhouette- and skeleton-based representations may be considered complementary approaches that capture different aspects of PD-related gait.

However, despite these advances, most existing studies remain limited to controlled laboratory settings or focus on a single task, such as distinguishing PD patients from healthy controls. Few investigations have addressed multiple clinically meaningful objectives simultaneously, including disease screening, functional assessment, and fall risk classification. In addition, many approaches rely on costly hardware or complex acquisition procedures, limiting their scalability and real-world applicability. Consequently, a gap persists between advances in computer vision and their translation into accessible clinical tools. To address these challenges, this study proposes a silhouette-based gait analysis framework for Parkinson’s disease. By analyzing consecutive silhouette images, the proposed framework captures global body shape and temporal movement patterns while minimizing identifiable appearance information. The proposed system aims to support clinically meaningful applications, including disease screening, functional assessment, and fall risk classification, thereby enabling scalable, objective, and home-based monitoring.

## Methods

### Study design and participants

This study is a cross-sectional observational study, in which gait data were collected from participants at a single time point under standardized conditions. All procedures were conducted in accordance with the Declaration of Helsinki and were approved by the Institutional Review Board of Show Chwan Memorial Hospital (Approval No. 1140207). All participants received a detailed explanation of the study purpose, procedures, and potential risks and benefits before participation. Written informed consent was obtained from all participants before the initiation of data collection. Participants were recruited from the neurology outpatient clinic of a teaching hospital, including patients with mild-to-moderate PD and age-matched healthy controls (HC). The diagnosis of PD was confirmed by specialized neurologists. Inclusion criteria consisted of: (1) a clinical diagnosis of PD based on the United Kingdom Parkinson’s Disease Society Brain Bank criteria; (2) mild-to-moderate PD, defined based on the Hoehn and Yahr staging system, specifically including patients classified as stages I, II, and III; (3) the ability to walk independently without assistive devices; (4) sufficient cognitive function to cooperate with the assessment procedures. Exclusion criteria included: (1) A history of other neurodegenerative diseases or stroke; (2) Severe musculoskeletal disorders affecting gait; (3) Visual or vestibular impairments impacting balance. HC were required to have no history of neurological disorders, major gait abnormalities, or chronic conditions affecting mobility. All participants completed clinical assessments and gait data collection. Gait videos from 40 participants were recorded during off-medication states. Detailed demographic information of the participants was presented in Table 2.

**Table 2:** Participant demographics.

	PD group (*n* = 29)	HC group (*n* = 11)	*p* value
Age	64.76 (8.34)	63.82 (6.01)	0.757
Gender, male	16 (55.2)	2 (18.2)	0.073
Duration of PD (years)	7.45 (4.46)	N. A.	
Hoehn and Yahr stages (on)	2.34 (0.40)	N. A.	
Hoehn and Yahr stages (off)	2.62 (0.73)	N. A.	

PD, Parkinson’s disease; HC, Healthy controls; N. A., not applicable.

### Clinical motor and functional assessment

Patients underwent concurrent clinical motor assessments during gait analysis. Motor severity was evaluated using Part III of the Unified Parkinson’s Disease Rating Scale (UPDRS Part III), with all evaluations independently performed by two experienced neurologists specializing in movement disorders. In addition to motor scoring, functional independence was assessed using the Schwab and England Activities of Daily Living (S&E ADL) scale (21). This scale provides a percentage score ranging from 0 to 100% in 10% increments; higher scores indicate a greater level of independence in daily activities. For the deep learning models, these clinical scores served as the ground-truth labels for model training and classification.

### Experimental environment and data acquisition

Gait data were recorded indoors within a standardized, well-lit capture area spanning approximately 500 cm in width under standard corridor fluorescent lighting. Participants performed structured mobility assessments consisting of a standard walking test and the Timed Up and Go (TUG) test. Participants completed five structured round-trip walking trials over a distance of approximately 360 cm, starting from a fixed position to ensure inter-trial consistency. An additional 55 cm was allocated at the starting boundary for a standardized chair setup used during TUG testing. For data collection, three synchronized iPhone 13 Pro Max cameras were used to record frontal, lateral, and oblique views, providing a comprehensive research video dataset. Videos were recorded at 30 frames per second (fps) with a resolution of 1080p (1920 × 1,080 pixels). The frontal camera was positioned approximately 460–560 cm in front of the walking trajectory, while the lateral and oblique cameras were positioned on the participant’s right side to capture profile and approximately 135° oblique views. All cameras were mounted approximately 130 cm above the floor. Although three views were recorded to establish a multi-view research dataset, only lateral-view videos were used for silhouette extraction and model analysis. The lateral view was selected *a priori* because sagittal-plane recordings facilitate the assessment of clinically relevant gait characteristics, including step length, gait speed, limb motion, and postural changes. Previous video-based gait studies have also demonstrated the utility of lateral-view smartphone recordings for gait assessment in individuals with PD (18). Accordingly, a standardized single-camera lateral-view protocol was adopted in this study.

### Video preprocessing and deep learning pipeline

The system utilizes an end-to-end deep learning pipeline to transform raw multi-view footage into standardized inputs and extract high-level feature representations. This pipeline operates in three distinct phases: [Raw Multi-View Video] → [YOLOX Object Detection] → [Deep Learning Silhouette Extraction] → [Sliding Window Segmentation].

#### Input data definition

Given a gait video, it is defined as a sequence of continuous RGB image frames:


$$V={\left\{I_{t}\right\}}_{t=1}^{T_{v}},I_{t}\in R^{H_{0}\times W_{0}\times 3}$$

where $T_{v}$ denotes the total number of frames in the video, and $H_{0}$ and $W_{0}$ represent the height and width of the original image, respectively.

#### Deep learning-based object detection and Silhouette extraction

To isolate dynamic gait characteristics and eliminate static environmental or background noise, a sequential computer vision pipeline was implemented. First, frame-by-frame pedestrian detection was performed using the anchor-free YOLOX deep learning architecture to isolate stable regions of interest (ROIs) (22). A pretrained YOLOX model was leveraged to guarantee robust feature extraction and continuous tracking of the participant across adjacent video frames.

Following ROI detection, pixel-level human segmentation was performed within each bounding box using PaddleSeg (23), which provides an effective balance between inference efficiency and segmentation accuracy. For the *t*-th video frame, the ROI $I_{t}[b_{t}]$ defined by the detected bounding box was extracted and processed by the segmentation model to generate a pixel-level probability map $\left(M_{t}\right)$ :

$$M_{t}=\text{PaddleSeg}(I_{t},b_{t})\in {\left[0,1\right]}^{H_{0}\times W_{0}}$$

The probability map was subsequently converted into a binary silhouette $(S_{t})\;$ by applying a predefined threshold τ using an indicator function:


$$S_{t}=\prod (M_{t}>\tau )$$

The silhouette representation reduces gait-irrelevant background, clothing texture, color, and other appearance-related information, thereby minimizing directly identifiable visual features and potential interference from cross-domain appearance variations while preserving overall body contour and gait-related movement patterns. To further evaluate the effect of gait representation on PD classification, an additional comparison between silhouette- and skeletal image-based inputs was conducted using the same PD/HC dataset, model architecture, and subject-independent data partitioning strategy. Details of the skeletal image-based approach are provided in Supplementary methods S1.

#### Sliding window silhouette clips generation

To focus on gait motion information and eliminate interference from physical appearance, the system first extracts a silhouette mask $I_{t}$ for each individual frame ${\tilde{S}}_{t}\in {\left[0,1\right]}^{H\times W}$ . Subsequently, a sliding window mechanism is employed to organize consecutive silhouette frames into fixed-length temporal clips:


$$C_{k}={\left\{{\tilde{S}}_{t}\right\}}_{t=t_{k}}^{t_{k}+T-1}\in R^{T\times H\times W\times 1}$$

where T represents the temporal length (frame depth) of a single clip, and K denotes the clip index. In this study, continuous silhouette sequences were partitioned using a fixed clip length (T) of 60 frames and a stride (K) of 60 frames. This specific hyperparameter configuration resulted in strictly non-overlapping windows (0% overlap), with each independent clip capturing approximately 2 s of continuous gait data at 30 fps. Because individual video recordings were bounded by the completion of the walking trials, the duration of each walking segment varied across participants, resulting in differences in the number of clips generated per segment. Most participants completed approximately five full walking rounds, with each round comprising two walking segments (left-to-right and right-to-left transits). To examine the potential contribution of walking speed and variation in clip numbers to PD/HC classification performance, additional walking-speed-based classification and clip-number standardization analyses were performed. Detailed procedures are provided in Supplementary methods S2.

### Multi-task deep learning framework

#### System architecture

The overall framework follows a modular, lightweight Multi-Task Learning (MTL) architecture optimized for edge deployments and home-based clinical screening (see Figure 1). The system consists of a shared front-end for image preprocessing, a unified spatiotemporal feature extraction backbone, and independent task-specific back-end prediction heads.

**Figure 1:**
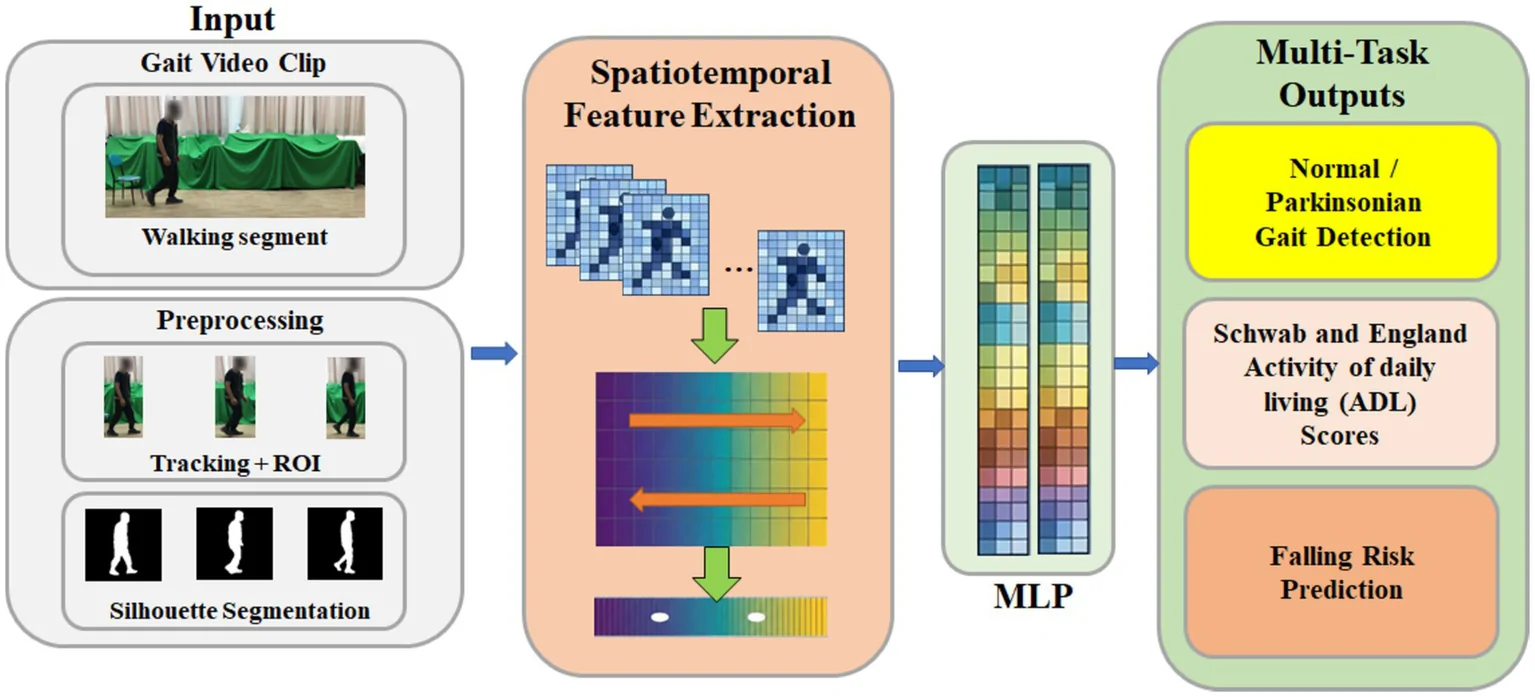
The proposed framework: the silhouettes are extracted from gait videos.

#### Spatiotemporal feature extraction

MoViNet (24) is employed as the shared deep 3D convolutional neural network (3D CNN) backbone network to extract high-dimensional spatiotemporal feature representations at the clip level for each silhouette $C_{k}$ . The clip-level spatiotemporal information is extracted by MoViNet as follows:


$$z_{k}=f_{\text{MoviNet}}(C_{k})\in R^{d}$$

where $f_{\text{MoviNet}}(.)$ denotes the MoViNet model function, and d represents the dimensionality of the feature vector.

#### Task-specific output heads and definitions

Based on the extracted spatiotemporal feature $z_{k}$ , this study developed dedicated recognition subsystems to address three core clinical tasks: Screening → Function → Risk. Each task is modeled with its own optimization objective:

1 *Screening task*: The PD screening system is formulated as a binary classification problem. The objective is to automatically determine whether a subject belongs to the “HC” group or the “PD” group. The system output label is denoted as $y^{\left(\mathrm{ΡD}\right)}\in [0,1]$ , which serves to assist in clinical early-stage screening. Its definition is as follows:
$$y^{\left(\mathrm{PD}\right)}\in [0,1]$$

2 *Functional assessment task*: The primary objective of the motor function assessment system is to predict the patient’s Activities of Daily Living (ADL). The system’s predicted value, $y^{\left(\mathrm{ADL}\right)}\in [80\%,90\%,100\%]$ , is aligned with the clinically recognized Schwab & England ADL (S&E-ADL) scale (21) to objectively reflect the patient’s actual quality of life. The value range is defined as follows:
$$y^{\left(\mathrm{ADL}\right)}\in [80\%,90\%,100\%]$$

(representing the percentage of functional ability, where 100% denotes full independence and 0% denotes complete dependence).

3 *Fall risk classification task*: The fall risk classification system is defined as a binary classification task focusing on assessing the fall risk of patients within a specific follow-up period. It identifies whether a subject belongs to a high-risk group with a propensity for falling based on retrospective self-reported data (at least one fall within the past year). The system identifies whether a subject belongs to the high-risk group with a propensity for falling ( $y^{\left(\text{Fall}\right)}\in [0,1]$ ), facilitating early preventative intervention in clinical settings. The output label for risk assessment is defined as follows:
$$y^{\left(\text{Fall}\right)}\in [0,1]$$

(where 0 represents low fall risk/non-fall, and 1 represents high fall risk/fall)

### Deep learning training and validation strategy

#### Training and optimization strategy

The proposed multi-task deep learning framework was developed and trained on a workstation equipped with an AMD Ryzen Threadripper PRO 7965WX processor, 320 GB RAM, and an NVIDIA RTX Pro 6,000 GPU. The framework was implemented in Python using TensorFlow, OpenCV, and NumPy. MoViNet-A0 (24, 25) was adopted as the spatiotemporal feature extraction backbone for all three tasks. For each task, the features extracted by MoViNet-A0 were passed to a task-specific classification head. As illustrated in Figure 2, the classification head consisted of a 1 × 1 × 1 Conv3D layer followed by a Swish activation function, a second 1 × 1 × 1 Conv3D layer for task-specific output mapping, and a squeeze operation to generate the final output. Separate classification heads were used for PD/HC classification, functional assessment, and fall history classification. Sparse categorical cross-entropy was used as the loss function for all three classification tasks. The models were trained using the Adam optimizer with a learning rate of 5 × 10^−5^, a batch size of 8, and 10 training epochs. No additional class-imbalance handling strategy, such as class weighting or oversampling, was applied during model training. The detailed training configuration is summarized in Table 1.

**Figure 2:**
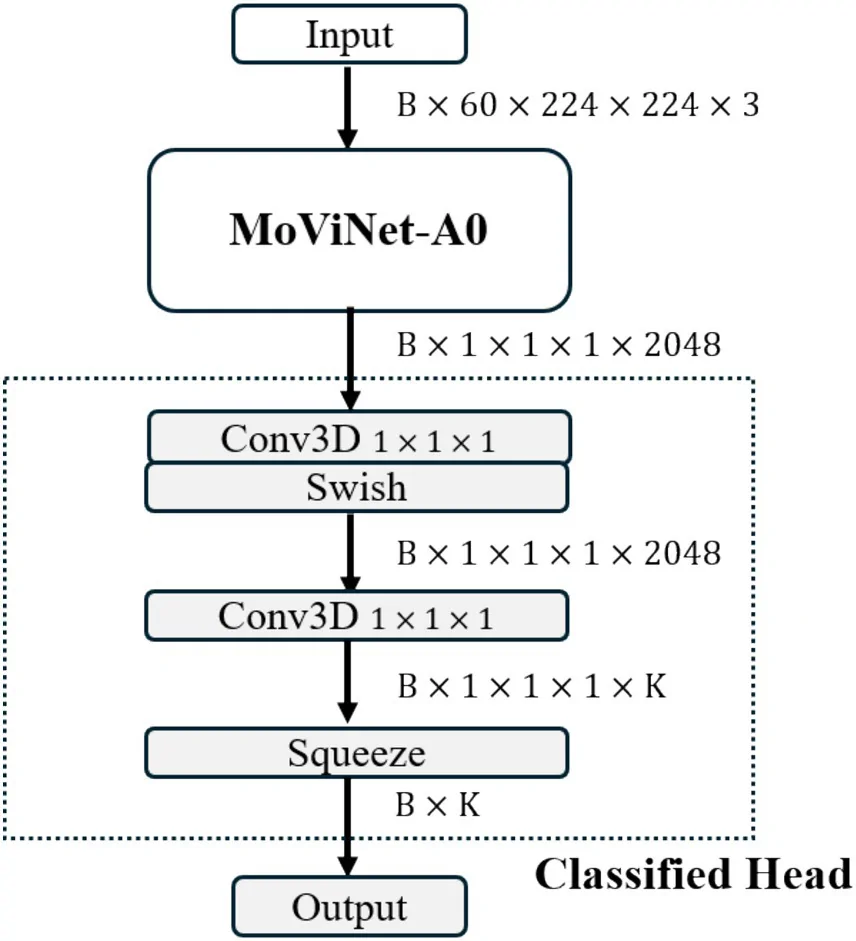
The architecture of the proposed deep learning models for three tasks.

**Table 1:** Training setup of the proposed models.

Training parameters	Value
Epochs	10
Optimizer	Adam
Learning rate	5e-5
Loss function	SparseCategoricalCrossentropy
Batch size	8

#### Data partitioning, validation, and subject-level evaluation

A subject-independent data partitioning strategy was implemented to ensure rigorous model evaluation and prevent data leakage. Data from each participant were assigned exclusively to either the training or testing set, with no overlap between sets. Furthermore, a cross-site validation protocol was used to assess the generalizability and stability of the trained deep learning framework across distinct physical environments and institutional data sources. To ensure consistency in performance evaluation, the final framework metrics, including accuracy, sensitivity, specificity, and the area under the receiver operating characteristic curve (AUC), were calculated based on participant-level aggregated predictions. Because the deep learning network performed inference on individual 2-s silhouette clips ( $C_{k}$ ), a decision-aggregation strategy was implemented to convert clip-level outputs into participant-level predictions. For the binary PD/HC and fall history classification tasks, majority voting across all clips belonging to each participant was used to determine the final classification. For the functional assessment task, clip-level predictions were averaged to determine the participant-level functional independence score.

#### Comparison of gait representations

To evaluate the effect of gait representation on PD/HC classification, an additional experiment was conducted comparing silhouette- and skeletal image-based inputs using the same MoViNet deep learning architecture. Both representations were derived from the same lateral-view videos and evaluated under identical experimental conditions, including the same subject-independent data partitions, frame sequence length, model configuration, and evaluation metrics. This comparison was designed to assess whether preserving the overall body contour in silhouette sequences provides additional discriminative information compared with skeletal image representations.

### Statistical analysis

Statistical analyses were performed to evaluate the demographic characteristics and the performance of the proposed models. Continuous variables are expressed as means and standard deviations (SD), while categorical variables are presented as absolute numbers and percentages. To compare continuous variables between groups (e.g., PD vs. HC), the Mann–Whitney U test was utilized. The Chi-square test (or Fisher’s exact test where appropriate) was applied for the comparison of categorical variables. To evaluate the predictive performance of the deep learning models across the classification tasks, receiver operating characteristic (ROC) curves were constructed, and the area under the curve (AUC) was calculated. These model evaluation metrics, including the mathematical computation of the ROC curves and AUC values, were executed programmatically using the scikit-learn package (version 1.7.2). All other baseline clinical and demographic statistical computations were executed using SPSS Statistics (Version 24.0; IBM Corp., Armonk, NY, USA). A significance threshold of *p* < 0.05 was adopted to determine statistical significance.

## Results

A total of 40 participants were enrolled from the neurology outpatient clinic of a teaching hospital, including 29 patients with PD and 11 HC (Table 2). The mean age of the PD group was 64.76 ± 8.34 years, compared with 63.82 ± 6.01 years in the HC group, with no significant difference between groups. In the PD group, 55.2% were male (*n* = 16), whereas 18.2% of the HC group were male (*n* = 2). Although the proportion of males was higher in the PD group, the difference did not reach statistical significance. Among PD participants, the mean disease duration was 7.45 ± 4.46 years. The mean Hoehn and Yahr stage was 2.34 ± 0.40 during the “on” state and 2.62 ± 0.73 during the “off” state, indicating that most patients were in the early to moderate stages of disease severity.

### Classification of PD and HC

In the training workflow of the first task, we integrated the GAIT-IT (26), GAIT-IST (27), and KOA-PD-NM (28) datasets with hospital-collected data and self-recorded videos. After image processing, a total of 2,713 training samples and 660 test samples were obtained. This study evaluated the model’s classification performance on both the training and independent test sets. The confusion matrix for the independent clinical test set showed that the model achieved an accuracy of 92.2% in the HC group, correctly classifying 59 of 64 samples, with only five samples misclassified. In the PD group, 252 of 261 samples were correctly classified, while only nine samples were misclassified as HC. These findings demonstrate the model’s strong ability to distinguish gait characteristics between PD and HC individuals in an independent clinical cohort (Figure 3a). Overall, the model achieved an accuracy of 95.7%, a sensitivity of 96.6%, a specificity of 92.2%, and an AUC of 0.994 on the independent clinical test set (Figure 3b). Detailed performance metrics are presented in Table 3.

**Figure 3:**
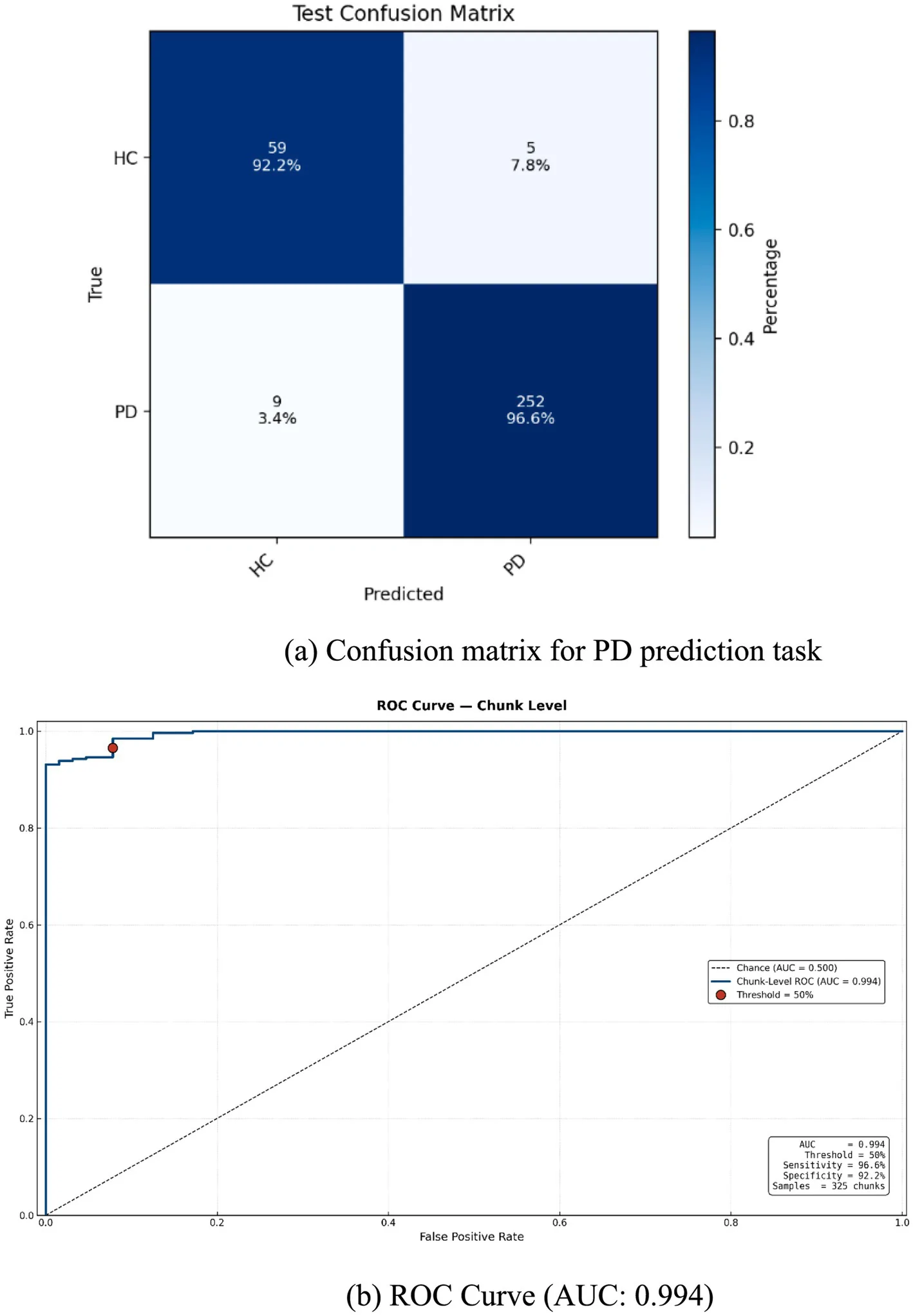
Confusion matrix **(a)** and ROC Curve **(b)** of the PD prediction task.

**Table 3:** PD identification task results.

	Accuracy (%)	Precision (%)	Recall (Sensitivity) (%)	F1 score (%)	Specificity (%)
Testing	95.7	98.1	96.6	97.3	92.2

Additional analyses were conducted to examine the potential contribution of walking speed to PD/HC classification. Participants with PD showed significantly lower walking speeds than HCs (0.952 ± 0.246 vs. 1.242 ± 0.240 m/s, *p* = 0.036). However, the walking-speed-only classifier showed substantially lower classification performance than the silhouette-based model (AUC = 0.724 vs. 0.994). Furthermore, after standardizing the number of clips between the PD and HC groups, the model maintained comparable classification performance (accuracy = 96.3%, AUC = 0.994). Detailed results are provided in Supplementary Tables S1–S3.

To further evaluate the effect of gait representation, an additional analysis was conducted to compare silhouette- and skeletal image-based representations for PD/HC classification under identical experimental conditions. The silhouette-based model achieved an accuracy of 95.7%, compared with 94.6% for the skeletal image-based model, demonstrating comparable classification performance between the two representations (Supplementary Table S4). Representative raw gait frames and their corresponding silhouette- and skeletal image-based representations are shown in Supplementary Figure S1.

As shown in Figure 4, representative raw walking images and their corresponding segmented silhouettes are presented to illustrate the preprocessing results of the proposed framework. The examples demonstrate variations in body posture and limb configurations across consecutive frames and individuals, providing a qualitative representation of walking characteristics.

**Figure 4:**
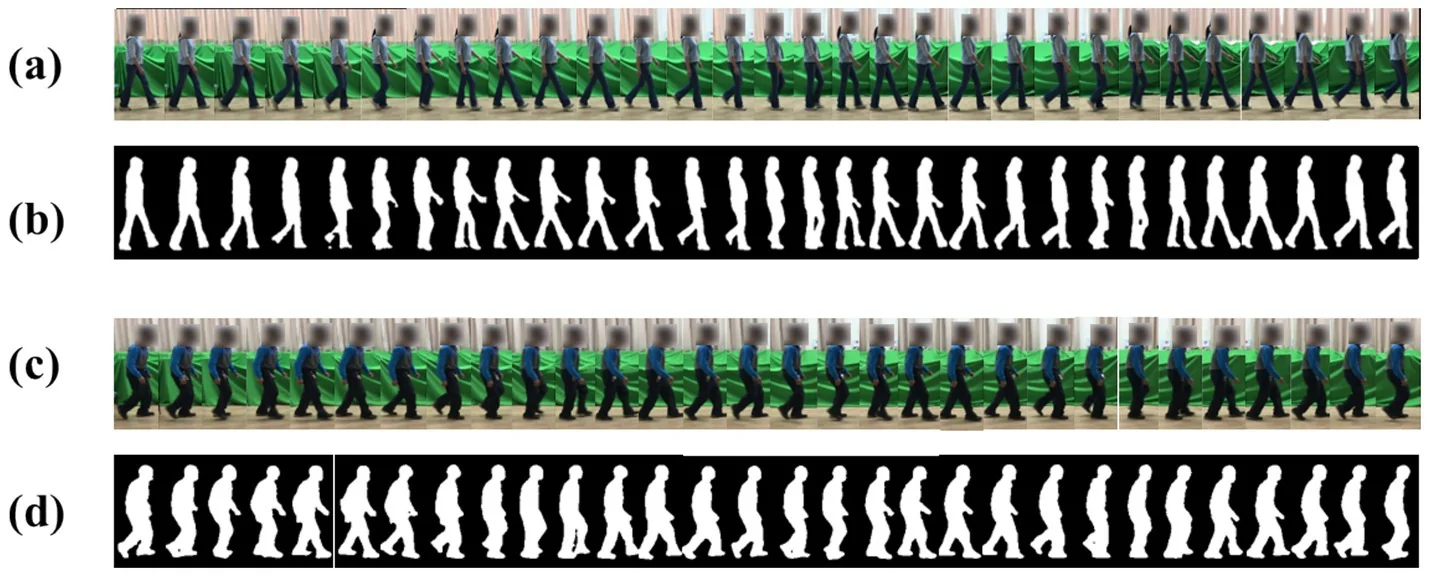
Representative walking image sequences and corresponding extracted gait silhouettes for visual illustration of input modalities. **(a)** Original video frames of an HC individual. **(b)** Segmented silhouette sequence of an HC individual. **(c)** Original video frames of a PD patient. **(d)** Segmented silhouette sequence of a PD patient. The sequences qualitatively illustrate distinct variations in posture and step distribution.

### ADL functional grading

After excluding samples with S&E ADL scores of 50 and 70%, the classification analysis was conducted across three functional categories (80, 90, and 100%). On the independent test set, the model achieved an overall accuracy of 88.5%, with a macro-average precision of 88.2%, a macro-average recall (sensitivity) of 87.0%, a macro-average F1 score of 87.5%, and a macro-average specificity of 94.1% (Table 4). Class-wise sensitivities were 100.0, 84.4, and 76.7% for the 80, 90, and 100% groups, respectively. All 42 samples in the 80% group were correctly classified. In the 90% group, 27 of 32 samples were correctly classified, while five samples (15.6%) were misclassified as 100%. In the 100% group, 23 of 30 samples were correctly classified, with six samples (20.0%) misclassified as 80% and one sample (3.3%) misclassified as 90% (Figure 5).

**Table 4:** ADL identification task results.

	Accuracy (%)	Macro-average precision (%)	Macro-average recall (Sensitivity) (%)	Macro-average F1 score (%)	Macro-average specificity (%)
Testing	88.5	88.2	87.0	87.5	94.1

ADL identification was a three-class classification task; therefore, Precision, Recall, F1 Score, and Specificity are reported as macro-averaged metrics across the three classes.

**Figure 5:**
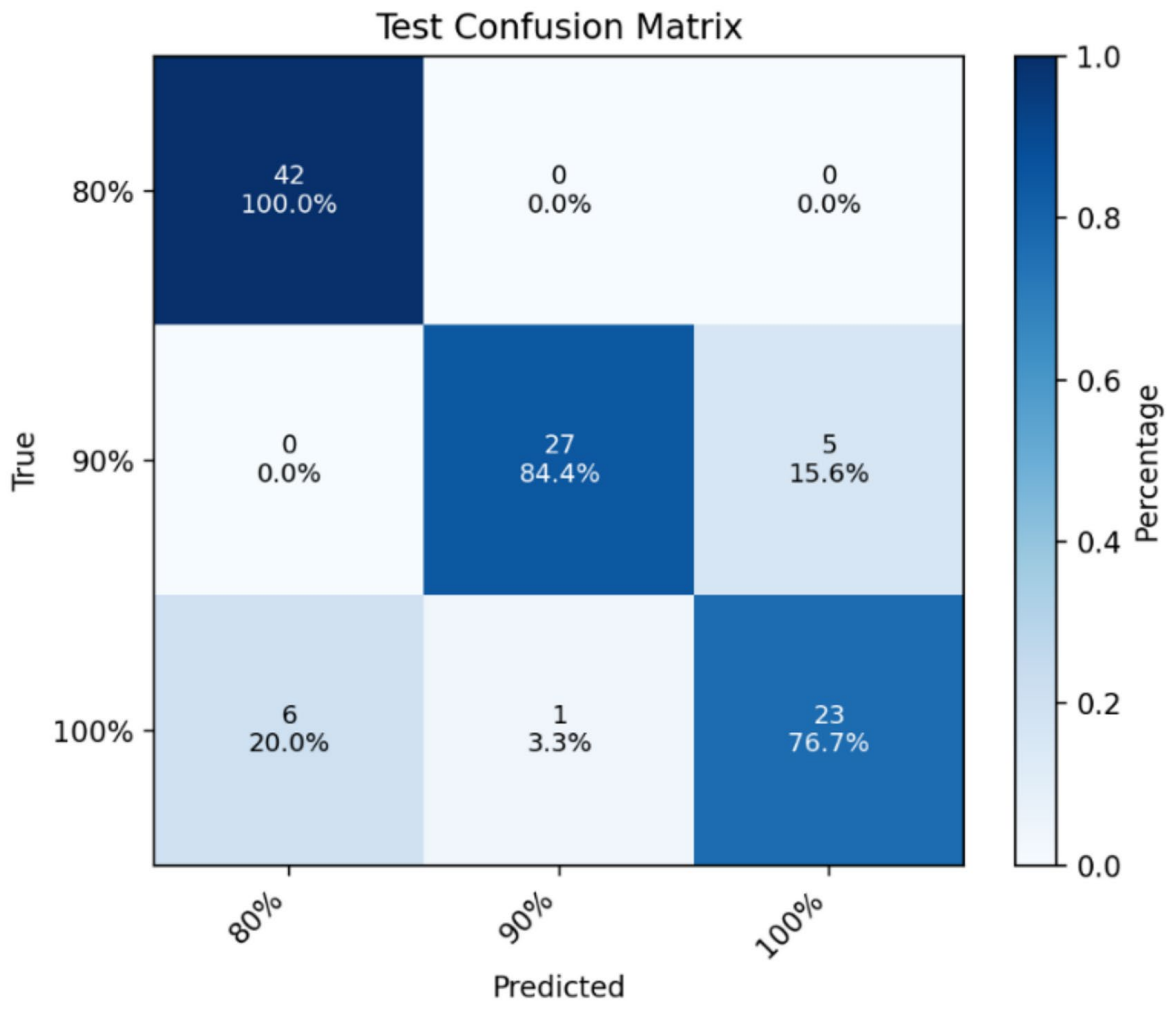
Confusion matrix for the ADL prediction task.

Table 5 presents the class-specific performance of the ADL identification task using a one-vs-rest evaluation strategy. The model achieved the highest performance in distinguishing the 80% ADL group from the other categories, with an accuracy of 95.2%, a sensitivity of 100.0%, a specificity of 91.9%, and an AUC of 0.969. For the 90% ADL group, the model attained an accuracy of 94.2%, a precision of 96.4%, a sensitivity of 84.4%, a specificity of 98.6%, and an AUC of 0.928. The 100% ADL group showed comparatively lower sensitivity (76.7%) and F1 score (79.3%), although the model still achieved an accuracy of 88.5%, a specificity of 93.2%, and an AUC of 0.960. Overall, the AUC values ranged from 0.928 to 0.969 across all ADL categories, indicating strong discriminative ability in identifying different levels of functional status.

**Table 5:** Classification performance for each ADL category (one-vs-rest analysis) on the independent test set.

Testing	Accuracy (%)	Precision (%)	Recall (Sensitivity) (%)	F1 Score (%)	Specificity (%)	AUC
ADL 80% vs. Remaining Levels	95.2	89.4	100	94.4	91.9	0.969
ADL 90% vs. Remaining Levels	94.2	96.4	84.4	90.0	98.6	0.928
ADL 100% vs. Remaining Levels	88.5	82.1	76.7	79.3	93.2	0.960

Performance metrics were calculated using a one-vs-rest evaluation strategy for each ADL category. AUC, area under the receiver operating characteristic curve.

As shown in Figure 6, representative walking image sequences and their corresponding silhouettes are presented for participants with S&E-ADL functional levels of 80, 90, and 100%. The examples illustrate variations in gait characteristics across different levels of functional ability, including body posture, step progression, and limb movement patterns, providing a qualitative representation of walking characteristics across functional levels.

**Figure 6:**
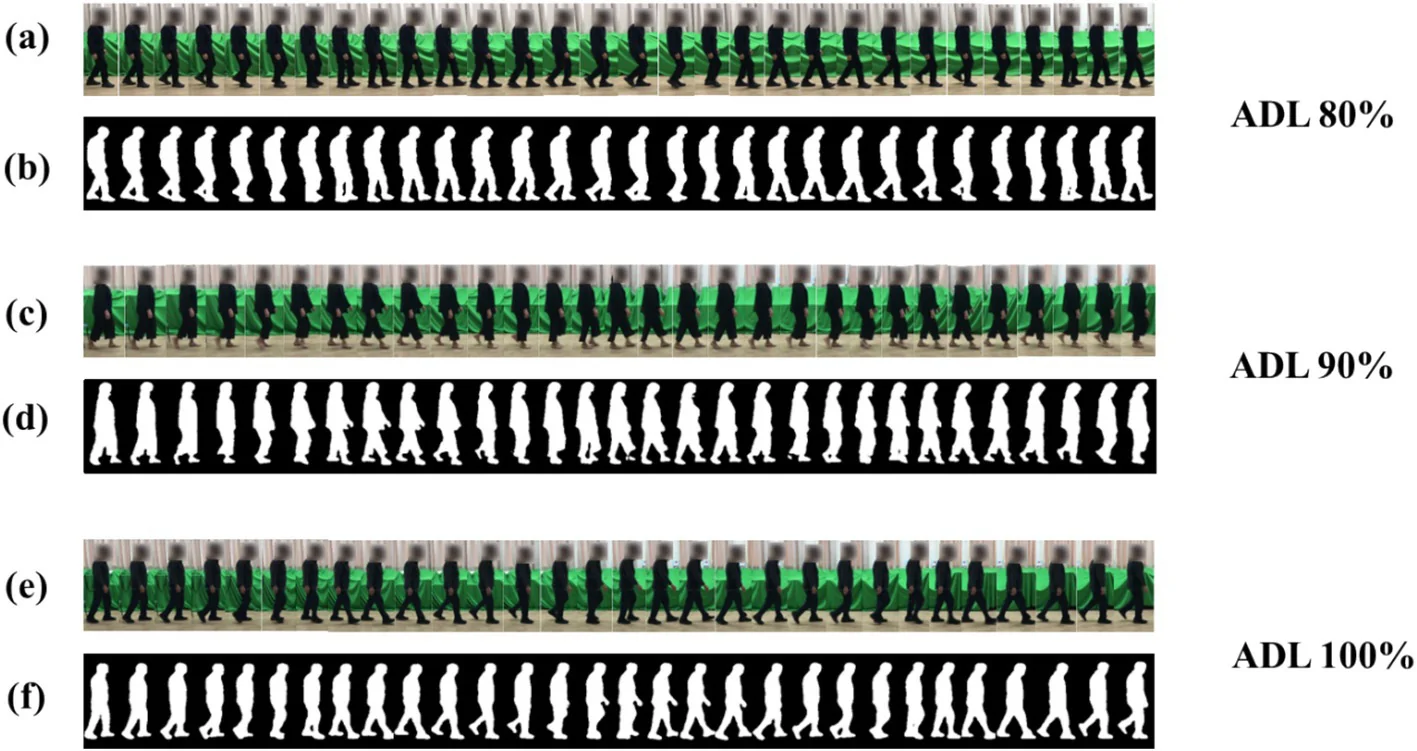
Representative walking image sequences and corresponding extracted gait silhouettes across different functional ability tiers for visual illustration. Panels **(a,c,e)** show the original walking image sequences, while panels **(b,d,f)** present the binarized silhouette sequences obtained through the image segmentation pipeline. These panels qualitatively demonstrate descriptive variations in dynamic contours and step progression.

### Fall risk classification

For Task 3 (fall risk classification), only PD participants were included in the analysis. On the independent test set, the model achieved an accuracy of 91.1%, a sensitivity of 68.4%, a specificity of 98.3%, and an AUC of 0.909. The confusion matrix showed that 118 of 120 non-fall samples and 26 of 38 fall samples were correctly classified, corresponding to a specificity of 98.3% and a sensitivity of 68.4%, respectively (Figure 7a). The ROC analysis yielded an AUC of 0.909 (Figure 7b). Detailed performance metrics are summarized in Table 6.

**Figure 7:**
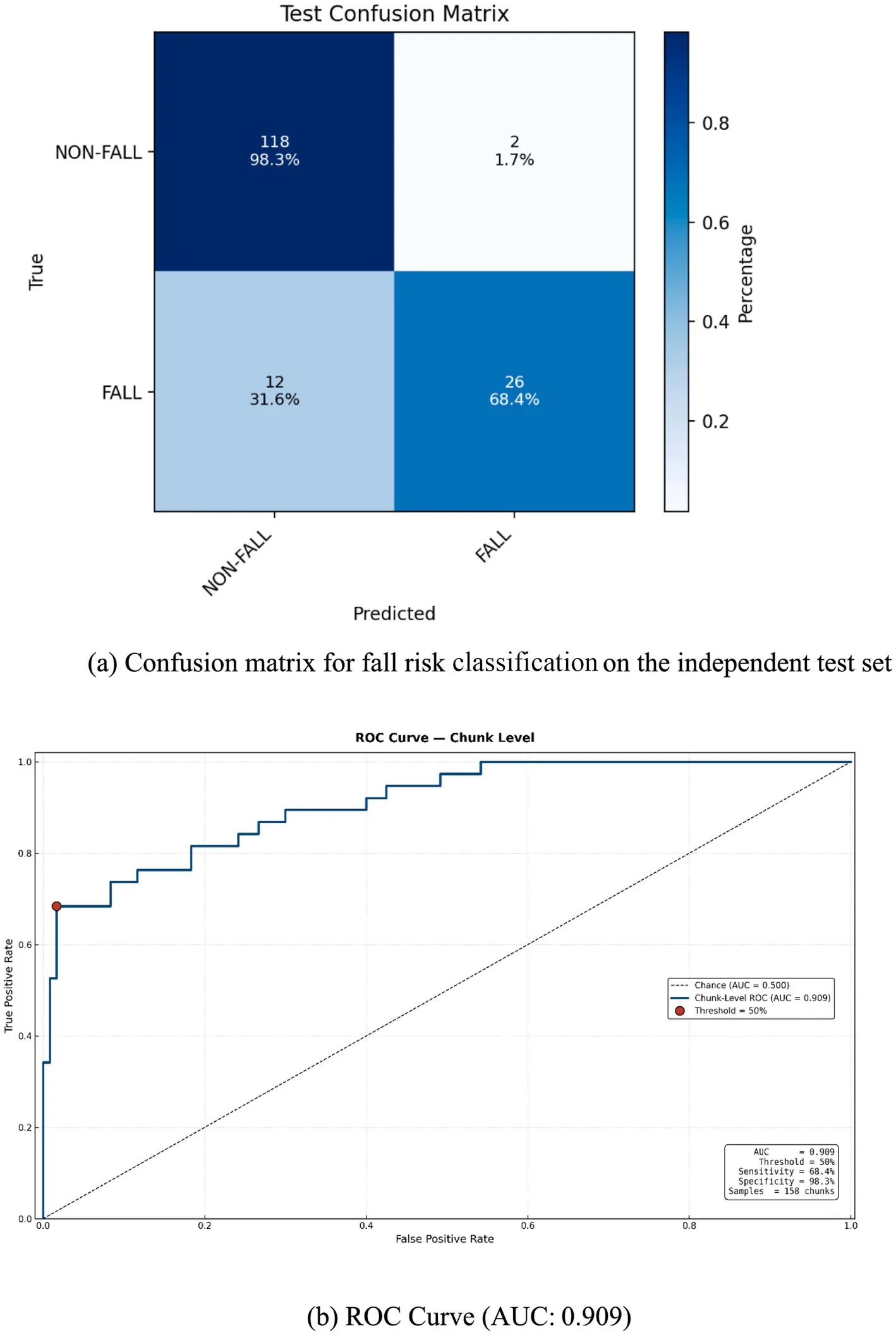
Confusion matrix **(a)** and ROC Curve **(b)** of the fall risk classification task.

**Table 6:** Fall risk classification task results.

	Accuracy (%)	Precision (%)	Recall (Sensitivity) (%)	F1 Score (%)	Specificity (%)
Testing	91.1	92.9	68.4	78.8	98.3

As shown in Figure 8, representative walking image sequences and their corresponding silhouettes are presented for participants with different fall-risk histories. The examples illustrate variations in gait characteristics, including body posture, step progression, limb positioning, and overall movement patterns, providing a qualitative representation of walking movements.

**Figure 8:**
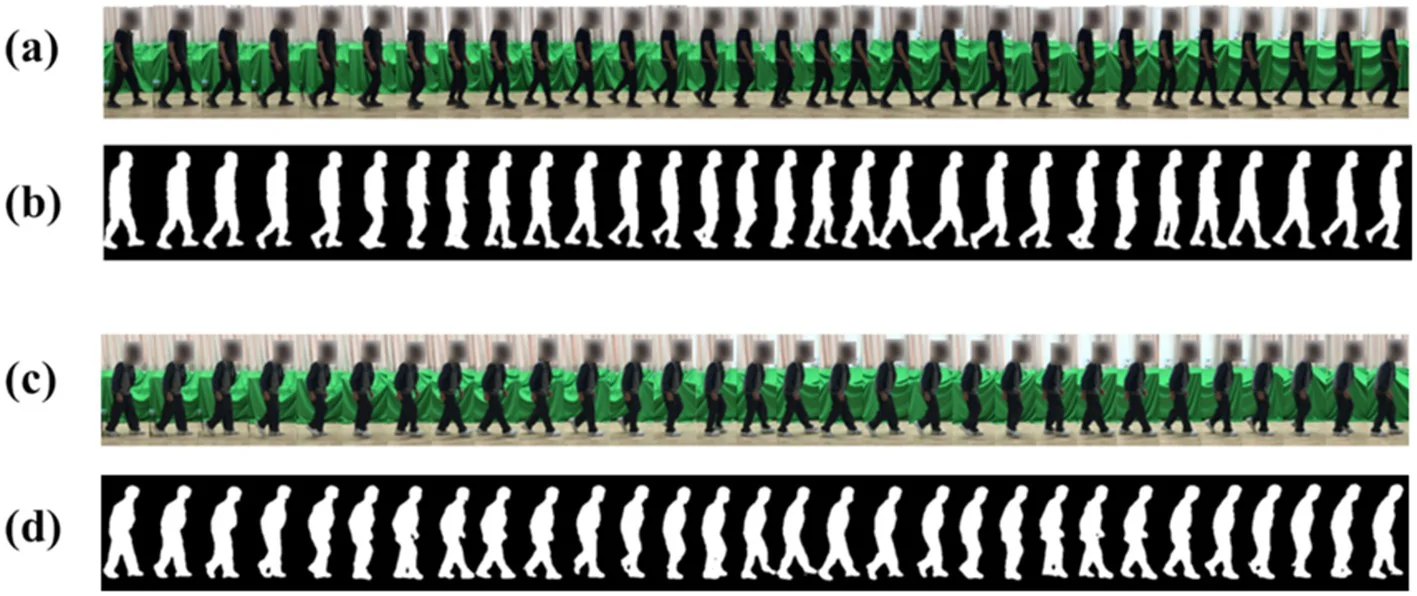
Representative walking image sequences and corresponding extracted gait silhouettes across different fall-risk clinical histories for visual illustration. Panels **(a,c)** show the original walking image sequences, while panels **(b,d)** present the binarized silhouette sequences obtained through the image segmentation pipeline. These panels qualitatively demonstrate descriptive variations in dynamic posture and step distribution.

It is important to emphasize that these visual observations across Figures 4, 6, 8 are intended strictly for qualitative illustration of the input data modality. Definitive clinical evaluation, disease classification, and functional grading are executed quantitatively by the downstream multi-task deep learning network rather than through subjective visual estimation. Collectively, these preliminary examples demonstrate that the silhouette extraction stage successfully isolates and preserves the broad spatiotemporal characteristics of gait while systematically removing background and appearance-related interference, thereby providing stable, thereby providing standardized visual input data for subsequent algorithmic analysis.

## Discussion

This study demonstrates the feasibility of utilizing video-based gait analysis as a non-invasive digital biomarker for PD, serving both as an objective clinical grading mechanism and with potential for future clinical and home-based monitoring applications (29, 30). Gait disturbance stands as one of the most prominent motor impairments in PD and remains a primary driver of functional disability and diminished quality of life (31). Within the broader elderly population, the prevalence of locomotion disorders escalates significantly with advancing age; approximately 35% of individuals aged 70 or older exhibit some degree of gait impairment (32), a figure that climbs sharply to 61.7% in cohorts over the age of 80 (33). Key gait characteristics such as walking velocity, cadence, stride length, and spatiotemporal variability have consistently proven to be powerful predictors of fall risks and overall functional decline (31, 34). Despite its immense clinical relevance, traditional gait assessment typically relies on specialized, expensive hardware (e.g., three-dimensional motion capture or computerized walkways) and time-consuming manual protocols, severely limiting its utility in routine clinical practice and continuous longitudinal monitoring (35).

### Clinical utility and framework innovations

Clinically, gait impairments in PD are categorized into continuous disturbances (e.g., reduced stride length, bradykinesia, and attenuated arm swing) and episodic disturbances (e.g., freezing of gait [FoG] and festination) (36). FoG manifests in approximately 50% of PD patients over the course of the disease and is characterized by a sudden, transient inability to initiate or continue stepping (37). These episodic phenomena are highly unpredictable and represent a leading cause of falls in PD populations (38). Resulting injuries frequently lead to emergency hospitalizations and long-term immobilization, significantly compounding healthcare expenditures and caregiver burdens. The primary innovation of this study lies in the construction of a wearable-free, vision-based deep learning gait analysis framework that operates entirely within natural environments (39). We validated its utility across three distinct clinical milestones: disease identification, functional stratification, and fall risk classification. Crucially, as detailed in our validation protocol, all evaluation metrics are aggregated and reported strictly at the participant level rather than the individual clip level. By using a majority voting ensemble for classification tasks and continuous prediction averaging for regression tasks, the framework bridges the gap between individual silhouette clip frames and meaningful, patient-centric clinical decisions. The framework utilizes segmented silhouette sequences to reduce background and appearance-related information while preserving overall body contours and temporal gait patterns (40).

Compared with skeletal representations, silhouettes retain global information on body shape, posture, and limb configuration without relying solely on estimated joint structures. To further examine the influence of gait representation, we implemented a skeletal image-based framework using YOLOX for participant localization (22), OpenPose for pose estimation (41), and MoViNet for spatiotemporal feature extraction (24). Skeletal representations preserve joint structure while reducing the influence of clothing, background, and appearance-related variations. Under the same experimental conditions, the silhouette-based and skeletal image-based models achieved accuracies of 95.7 and 94.6%, respectively, indicating comparable performance for PD/HC classification (Supplementary Table S4). However, the two representations capture different aspects of gait. Skeletal images provide explicit joint-level structural information, whereas silhouettes retain more complete information on body contour, posture, and limb configuration across consecutive frames. Therefore, these findings do not indicate the superiority of one representation over the other but rather suggest their potential complementary value for PD gait assessment.

While the representative images in Figures 4, 6, 8 qualitatively illustrate variations in posture, limb configuration, and gait patterns across clinical groups, model evaluation was based on quantitative classification performance rather than visual interpretation of these images. Overall, the proposed video-based framework reduces the need for wearable sensors or specialized gait-analysis equipment, thereby simplifying gait assessment.

### Task-specific performance and limitations

In the PD identification task, although the proposed framework achieved excellent performance during training, a decline in accuracy and sensitivity was observed on the independent test set. Similar findings have been reported in numerous medical AI studies, where models developed using one dataset frequently experience performance degradation when applied to data from different institutions or collection environments. Such reductions are often attributed to differences in participant characteristics, recording conditions, and underlying data distributions, highlighting the ongoing challenge of cross-dataset generalization and the need for further validation across diverse clinical settings (42). This gap likely stems from the highly subtle gait variations characteristic of early-to-mid-stage PD, high inter-individual variability, and environmental domain shifts within the test environment. Nevertheless, the model maintained robust specificity for healthy individuals, demonstrating reliable stability in excluding non-disease populations. Further improvements may be achieved through larger sample sizes, targeted data augmentation, and cross-site external validation.

Several limitations related to video acquisition should also be acknowledged. Although multi-view videos were acquired, only lateral-view videos were analyzed, and performance across camera views was not compared. In addition, the standardized single-camera setup, including fixed camera positioning, walking distance, and indoor lighting, may limit generalizability to unconstrained home environments. Future studies should evaluate view-specific performance and validate the framework under more diverse clinical and home-based conditions.

Regarding functional stratification, the proposed framework demonstrated the ability to distinguish among different S&E-ADL levels using deep spatiotemporal gait features. These findings suggest that video-derived gait patterns contain clinically relevant information associated with functional impairment and support the potential use of vision-based assessment as a non-invasive tool for monitoring disease-related functional decline in PD. The framework maintained superior classification capabilities at the functional extremes (e.g., 80% versus 100% ADL), though performance slightly softened at intermediate levels. This behavior reflects the continuous nature of the ADL scale, where intermediate functional boundaries naturally exhibit clinical feature overlaps.

In the fall risk classification task, while overall accuracy remained stable, the sensitivity for the fall-prone cohort was lower, indicating that certain high-risk phenotypes went undetected. Given that fall risk classification is a high-stakes clinical screening scenario, mitigating false negatives is paramount. Future structural enhancements will focus on adjusting loss function topologies via minority-class reweighting (e.g., focal loss), oversampling high-risk cohorts, or integrating multimodal signals to maximize clinical safety.

### Future directions and clinical translation

It is critical to acknowledge that falling is inherently a multifactorial phenomenon governed by complex interactions between environmental hazards, cognitive reserves, and motor control integrity. Clinical literature indicates that the perceived difficulty of ambulation in PD patients progressively worsens alongside disease severity. Personal psychological factors (such as fear of falling or hyper-vigilance) and objective motor stressors (such as postural instability under dual-task conditions) possess profound predictive value (43). Consequently, while single-modality vision features show immense promise, integrating cognitive loads, situational environmental variations, and psychological baselines will be instrumental in building a comprehensive, clinically dependable fall risk classification model.

For vision-based gait models to successfully transition from benchmarking datasets to real-world clinical deployment and smart-home integration, two primary considerations are relevant. First, the silhouette-based approach reduces facial, clothing, and other appearance-related information while preserving gait-related movement patterns, thereby providing a more standardized visual representation. Second, the framework supports cross-site domain generalization by reducing the influence of variations in camera specifications, lighting conditions, and background environments across institutional datasets. Future work should prioritize cross-site validation and unsupervised domain adaptation strategies to fortify system stability.

On a technical level, the use of MoViNet enables efficient spatiotemporal feature learning from gait sequences (24). Its specialized Stream Buffer mechanism enables the framework to conduct real-time inference on resource-constrained edge devices, significantly enhancing its deployment potential in mobile health (mHealth) and telecare settings. Ultimately, the proposed framework establishes a scalable foundation for non-invasive digital phenotyping. Future longitudinal tracking designs will enable this tool to monitor disease progression and evaluate objective rehabilitation outcomes, maximizing its practical utility in clinical translation.

## Conclusion

This study developed a smartphone-video-based gait assessment framework using computer vision and deep learning. The proposed framework demonstrated promising performance in identifying patients with PD, assessing functional status, and predicting fall risk using gait videos captured by smartphones. Future research will focus on expanding the sample size across diverse populations and disease stages to enhance the model’s generalization capabilities. Further development may incorporate multimodal information, such as skeletal features, speech patterns, and fine motor indicators, to improve the assessment of complex clinical manifestations such as FoG. Ultimately, integrating the framework into smartphone platforms with on-device inference may facilitate automated home-based screening and longitudinal monitoring of PD.

## Data Availability

The raw data supporting the conclusions of this article will be made available by the authors, without undue reservation.
