# Supplementary material for: A deep learning-based silhouette gait detection framework for fall risk classification in Parkinson’s disease
Source: Front Neurol. 2026 Sep 29;17:1944666. doi: 10.3389/fneur.2026.1944666 (PMC13660010; doi:10.3389/fneur.2026.1944666)
Supplement: Supplementary file 1 [file Data_Sheet_1.PDF]

Supplementary Material

1. Methods

S1. Skeletal Image-Based Framework for PD Classification

Skeleton Segmentation

In this study, the OpenPose model [S1] was used for human pose estimation. OpenPose employs Part Confidence Maps to localize body joints and Part Affinity Fields to establish connections between joints, enabling the detection of 18 skeletal keypoints. For the t-th frame, the skeleton-based analysis system extracted the ROI $I_t[b_t]$ defined by the bounding box and fed it into the OpenPose model to generate a joint keypoint map P_t:

$$P_t = OpenPose(I_t, b_t) = \{p_{t,j}\}_j^{18}$$

Where

$$p_{t,j} = \left(x_{t,j}, y_{t,j}, q_{t,j}\right),$$

$q_{t,j} \in [0, 1]$represents the confidence score of the j-th joint keypoint.

Subsequently, the RenderStick function was used to connect the detected joint keypoints with white lines, thereby converting the joint keypoint map into a binarized skeletal image BWP_t :

$$BWP_t = \text{RenderStick}(P_t)$$

The skeletal images retained key anatomical landmarks, including the neck, shoulders, elbows, wrists, pelvis, hips, knees, ankles, heels, and toes, to capture gait-related characteristics potentially associated with PD, such as reduced arm swing, forward trunk posture, short shuffling steps, foot dragging, and reduced lower-limb movement amplitude. The resulting skeletal image sequences were subsequently processed using MoViNet for spatiotemporal feature extraction, as described in the Spatiotemporal Feature Extraction subsection.

Supplementary Reference

1. Cao Z, Hidalgo G, Simon T, Wei SE, Sheikh Y. OpenPose: Realtime Multi-Person 2D Pose Estimation Using Part Affinity Fields. IEEE Trans Pattern Anal Mach Intell. 2021;43(1):172-86.

S2. Walking-Speed and Clip-Number Standardization Analyses

Walking-Speed Estimation and Speed-Based Classification

For participant p, walking speed during the r-th walking segment ($WS_{p,r}$) was calculated as follows:

$$WS_{p,r} = \frac{WD}{NFrame_{p,r} * (\frac{1}{fps})}$$

where WD represents the walking distance, which was 360 cm in this study. $NFrame_{p,r}$ represents the total number of frames recorded for participant p during the r-th walking segment; and fps represents the video frame rate, which was fixed at 30 frames per second. The mean walking speed was then calculated across all available walking segments for each participant. The mean walking speeds of the PD and HC groups are presented in Table S1.

After calculating the walking speed for each walking video, the corresponding walking speed was assigned to that video and used as the sole input feature to train a Random Forest classifier for PD/HC classification. The classification performance of the walking-speed-based Random Forest classifier was compared with that of the proposed silhouette-based abnormal gait recognition framework, as shown in Table S2.

Clip-Number Standardization

Because HC participants generally walked faster than PD participants, fewer clips were generated within the same fixed walking distance. To minimize this clip-number imbalance and assess the potential influence of walking-speed differences on PD/HC classification, the HC testing videos were resampled from 30 to 60 fps. The classification results after clip-number standardization are presented in Table S3.

2. Results

Table S1. Comparison of walking speed between the PD and HC groups

Group	Walking speed, mean (m/s)	SD (m/s)	p value
HC	1.242	0.240	0.036
PD	0.952	0.246	

Note. Walking speed was compared between the HC and PD groups using the Mann-Whitney U test. A p-value < 0.05 was considered statistically significant.

Table S2. Performance of the walking-speed-only classifier and the silhouette-based model for PD identification

Model	Accuracy (%)	Precision (%)	Recall (Sensitivity) (%)	F1 Score (%)	Specificity (%)	AUC
Walking speed-only	67.6	74.7	81.6	78.0	34.4	0.724
Silhouette	95.7	98.1	96.6	97.3	92.2	0.994

Note. The walking-speed-only classifier was developed using a Random Forest model with walking speed as the sole input feature. AUC, area under the receiver operating characteristic curve.

Table S3. Classification performance after clip-number standardization

Model	Accuracy (%)	Precision (%)	Recall (Sensitivity) (%)	F1 Score (%)	Specificity (%)	AUC
Silhouette	96.3	97.3	96.6	96.9	95.9	0.994

Table S4. Performance comparison between silhouette- and skeletal image-based models for PD identification

Model	Accuracy (%)	Precision (%)	Recall (Sensitivity) (%)	F1 Score (%)	Specificity (%)
Silhouette	95.7	98.1	96.6	97.3	92.2
Skeletal image	94.6	99.5	93.9	96.6	97.7

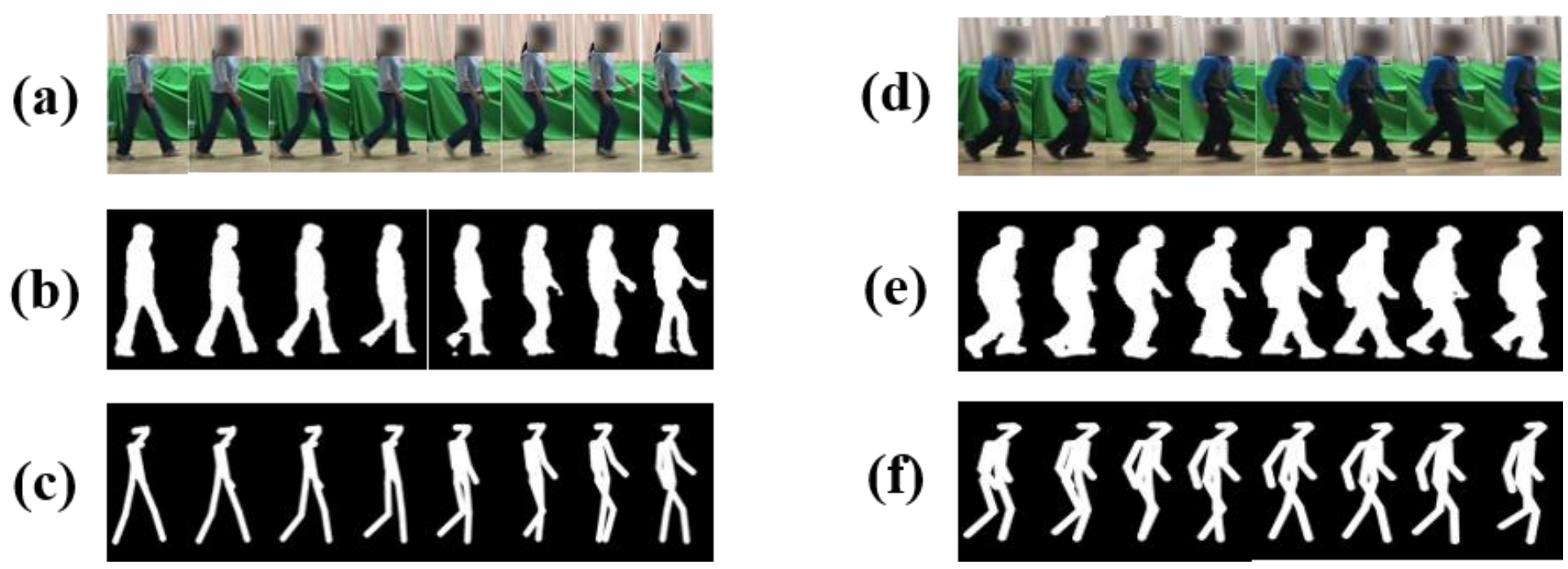

Figure S1. Representative gait image sequences and corresponding silhouette and skeletal representations. (a) Original video frames of an HC individual. (b) Segmented silhouette sequence of an HC individual. (c) Skeletal image sequence of an HC individual. (d) Original video frames of a PD patient. (e) Segmented silhouette sequence of a PD patient. (f) Skeletal image sequence of a PD patient. The figure illustrates the transformation of raw gait videos into silhouette- and skeleton-based representations used for subsequent spatiotemporal feature extraction.
